# Characterization of lncRNA-Perturbed TLR-Signaling Network Identifies Novel lncRNA Prognostic Biomarkers in Colorectal Cancer

**DOI:** 10.3389/fcell.2020.00503

**Published:** 2020-06-18

**Authors:** Yanjie Chu, Zhiqiang Liu, Jing Liu, Lei Yu, Dekai Zhang, Fenghua Pei

**Affiliations:** ^1^Department of Gastroenterology and Hepatology, The Second Affiliated Hospital of Harbin Medical University, Harbin, China; ^2^Department of Hematology, Harbin Medical University Cancer Hospital, Harbin, China; ^3^Department of Colorectal Tumor Surgery, The Second Affiliated Hospital of Harbin Medical University, Harbin, China; ^4^Center for Infectious and Inflammatary Diseases, Texas A&M University, Houston, TX, United States

**Keywords:** Toll-like receptors, long non-coding RNAs, colorectal cancer, ceRNA network, biomarkers

## Abstract

Increasing evidence has suggested that long non-coding RNAs (lncRNAs) are critical regulators in the Toll-like receptors (TLR)-signaling network to modulate colorectal cancer (CRC) development and progression. However, the mechanism and clinical significance for lncRNAs regulating TLR signaling pathways in CRC remained largely unknown. In this study, we performed an integrative network analysis of transcriptomics by focusing on a lncRNA-perturbed TLR-signaling network, identifying 280 lncRNAs and 122 mRNAs. We found a profound phenomenon that abnormal expression of some lncRNAs can perturb the TLR-signaling network to contribute to CRC development and progression. Furthermore, we identified a novel TLR-related prognostic gene signature (TLRLncSig) composed of three lncRNAs (*MCHR2*, *AC011472.4*, and *AC063944.1*), and one mRNA (*CDKN2B*). Utilizing TLRLncSig could classify CRC patients of training set into two groups with significantly different overall survival. The prognostic value of the TLRLncSig was further validated in the other two independent CRC datasets with different platforms. Results of multivariate and stratification analysis indicated that the TLRLncSig is an independent prognostic factor, and our study underscores the clinical significance of TLR-related lncRNAs in CRC development and progression.

## Introduction

Colorectal cancer (CRC) is the third most common form of cancer incidence and death in both men and women and ranked top 1 in digestive system cancers (Siegel et al., 2020a). It is estimated that there are 147,950 estimated new cases and 42,170 estimated deaths in the United States according to cancer statistics, 2020 (Siegel et al., 2020b). Surgery followed by chemotherapy and radiotherapy is the most common treatment. However, approximately 25–40% of patients develop tumor relapse (Walker et al., 2014). Although the classic staging system was commonly used, it is not sufficient for prognosis prediction because of intertumor and intratumor heterogeneity of cancer (Aziz et al., 2017). Therefore, molecular biomarkers were needed to identify for improving prognosis.

Toll-like receptors (TLRs) are key players of the innate immune system. The accumulating evidence suggests that TLRs play essential roles in the activation of innate immunity and the development of antigen-specific acquired immunity (Akira and Takeda, 2004). There is increasing evidence indicating that expression alteration in TLRs is associated with cancer development and can influence infection susceptibility. Differential expressions of TLRs and TLR-related protein have been observed in CRC compared with normal individuals, and are associated with patient survival (Khan et al., 2016; Bednarczyk et al., 2017). For example, higher expression of the TLR1, TLR2, TLR4, TLR8, and TLR9 genes were observed in CRC tissues when compared to normal colon tissues (Lu et al., 2014), highlighting the critical roles of TLR in CRC development and progression.

Long-coding RNAs (lncRNAs) were defined as RNA transcripts with little or no protein-coding capacity and greater than 200 nucleotides in length (Kung et al., 2013). Until now, tens of thousands of non-coding RNAs (ncRNAs) have been identified and predicted in various species through experimental and computational methods, and become a significant class of ncRNAs (Zhou et al., 2009, 2015a; Sun et al., 2014). Increasing functional studies have demonstrated that lncRNAs are emerging as master regulators involved in transcriptional and post-transcriptional regulation as well as chromatin remodeling by function as decoys, scaffolds, and enhancer RNAs (Fang and Fullwood, 2016). Recent studies have also revealed that lncRNAs are an essential part of the networks involved in regulating TLR-signaling pathways by function as competitive endogenous RNA (ceRNA). For example, lncRNA *XLOC_098131* was found to regulate the TLR-signaling pathways by serving as ceRNA to stabilize FOS mRNA expression via binging to *miR-548s* (Fan et al., 2019). Another lncRNA *SHNG16* was recently reported as a ceRNA to regulate TLR4 via competitively binding miR-15a/16 positively [15]. Despite the emerging role of lncRNAs as critical regulators in TLR-signaling pathways, the lncRNA-mediated regulatory mechanism in TLR-signaling pathways and their clinical significance in CRC remain mostly unknown.

In this study, we tried to construct a lncRNA-mediated TLR-signaling network involved in CRC development for exploring the potential involvement of lncRNAs in TLR-signaling pathways through integrative transcriptomics and network analysis in a large number of CRC samples. We further investigated and validated the prognostic value of lncRNAs involved in the TLR-signaling network in multiple CRC patient cohorts.

## Materials and Methods

### Clinical and Transcriptomics Data of CRC Patients

Clinical characteristics and transcriptomics data of CRC patients were obtained from the UCSC Xena platform^1^ and Gene Expression Omnibus database (GEO, https://xena.ucsc.edu/), including 427 CRC patients and 41 control samples from The Cancer Genome Atlas (TCGA), and 585 CRC patients from GSE39582^2^ (Marisa et al., 2013). The detailed information on clinical data of all CRC patients used in this study was summarized in Table 1.

**TABLE 1:** Clinical characteristics of CRC patients in each dataset used in this study.

Covariates		Training set (214)	Internal testing set (*n* = 213)	GSE39582 (*n* = 575)
Age, years (mean ± SD)		67.2 ± 12.1	65.5 ± 13.3	66.8 ± 13.2
Gender, no (%)	Male	120 (56.1)	111 (52.1)	317 (55.1)
	Female	94 (43.9)	102 (47.9)	258 (44.9)
Stage, no (%)	I	28 (13.1)	44 (20.7)	37 (6.4)
	II	84 (39.2)	79 (37.1)	266 (46.3)
	III	56 (26.2)	65 (30.5)	209 (36.3)
	IV	40 (18.7)	20 (9.4)	59 (10.3)
	Unknown	6 (2.8)	5 (2.3)	4 (0.7)
Pathological T	T0	0 (0.0)	0 (0.0)	1 (0.2)
	T1	3 (1.4)	7 (3.3)	12 (2.1)
	T2	34 (15.9)	41 (19.2)	48 (8.4)
	T3	147 (68.7)	145 (68.1)	374 (65.0)
	T4	30 (14.0)	19 (8.9)	117 (20.3)
	Unknown	0 (0.0)	1 (0.5)	23 (4.0)
Pathological N	N0	118 (55.1)	132 (62.0)	308 (53.6)
	N1	56 (26.2)	44 (20.6)	135 (23.5)
	N2	40 (18.7)	37 (17.4)	100 (17.4)
	N3	0 (0.0)	0 (0.0)	6 (1.0)
	Unknown	0 (0.0)	0 (0.0)	26 (4.5)
Pathological M	M0	148 (69.2)	168 (78.9)	493 (85.7)
	M1	40 (18.7)	20 (9.4)	60 (10.4)
	Unknown	26 (12.1)	25 (11.7)	22 (3.8)
Vital status, no (%)	Alive	160 (74.8)	176 (82.6)	385 (67.0)
	Dead	54 (25.2)	37 (17.4)	190 (33.0)
Relapse, no (%)	With	−	−	391 (68.0)
	Without	−	−	179 (31.1)
	Unknown	−	−	5 (0.9)

### Acquisition and Preprocessing of lncRNA Expression Profiles of CRC Patients

Raw RNA sequencing data (level 3) of CRC patients and control samples based on the IlluminaHiSeq_RNASeq platform from UCSC Xena were obtained and normalized to Fragments Per Kilobase of transcript per Million mapped reads (FPKM) values. A total of 14799 lncRNAs were obtained based on known lncRNA annotations in the GENCODE database^3^. Raw microarray data (.CEL files) of CRC patients on Affymetrix Human Genome U133 Plus 2.0 (Affymetrix HG-U133 Plus 2.0) from the GEO database were obtained and were processed and normalized using robust multichip average method through R “affy” package. A total of 3475 lncRNAs were obtained based on the NetAffx annotation files of the probe sets and known lncRNA annotations of RefSeq and GENCODE, according to previous studies (Zhou et al., 2018a).

### Analysis of lncRNA Expression Profiles of CRC Patients

Differentially expression analysis was performed using the R “DESeq2” package. Those genes or lncRNAs with a false discovery rate (FDR) <0.05 and | log2(fold change) >1.0 was identified as significantly differentially expressed. Hierarchical clustering of samples based on expression values of differentially expressed TLR-related genes was performed with R software using the metric of Euclidean distance and complete linkage.

### TLR-Related Genes

104 genes in the TLR pathway were obtained from the Kyoto Encyclopedia of Genes and Genomes (KEGG). Protein-protein interaction network (PPID) was obtained from STRING database^4^. 8611 directly interacting neighbors of 104 TLR genes were retrieved from PPID and defined as TLR-related genes.

### Statistical Analysis

Kaplan-Meier survival curves and log-rank tests were used to compare survival differences between high-risk group and low-risk group with the R package “survival.” Univariate and multivariate Cox proportional hazards regression analyses were conducted through the R package “survival.” Univariate and multivariate Cox regression analyses for OS were used to identify independent prognostic biomarkers. A risk score signature for each patient was constructed using the expression values of each prognostic biomarker, weighted by their estimated regression coefficients in the multivariate Cox regression analysis as previous studies. Patients were classified into the high-risk group or low-risk group according to the optimal risk cut off value derived from the R package “maxstat.”

## Results

### Identification of Key Genes Related to the TLR Pathway in CRC

**FIGURE 1:**
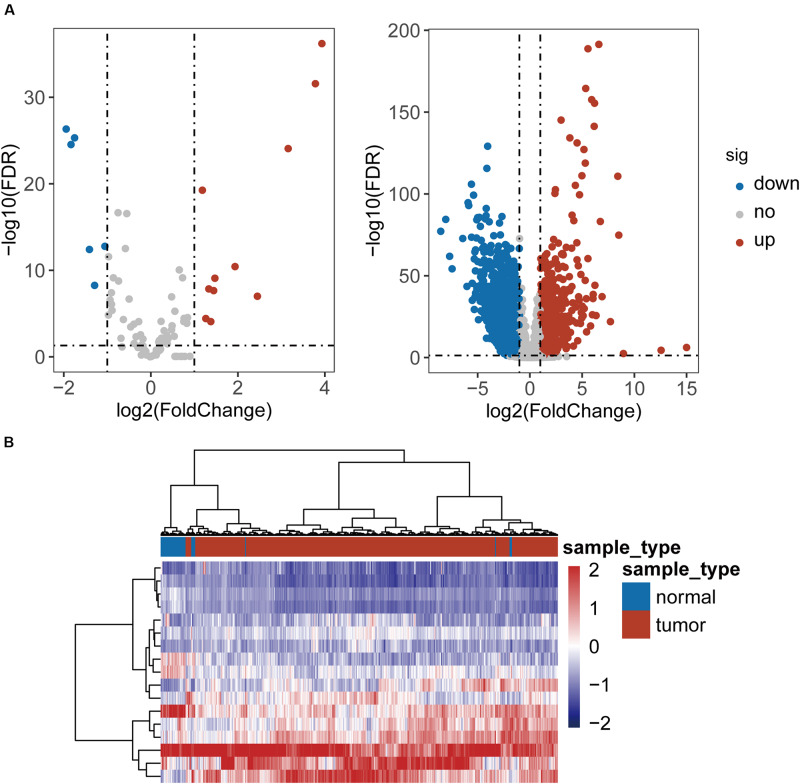
Differential expressed analysis of TLR-related genes between 41 paired CRC and control samples. **(A)** Volcano plot of the distribution of differentially expressed TLR-related genes. **(B)** Hierarchical clustering heatmap and dendrogram of 468 TCGA CRC samples based on differentially expressed TLR-related genes.

To identified TLR-related genes in CRC, we performed differentially expression analysis for 104 TLR genes and 8611 TLR-related genes between 41 paired CRC and control samples, and identified 17 differentially expressed TLR genes (including 11 up-regulated and 6 down-regulated in CRC) and 2338 differentially expressed TLR-related genes (including 879 up-regulated and 1459 down-regulated in CRC) [|log2(fold change)| > 1 and FDR-adjusted *p*-value < 0.05] (Figure 1A and Supplementary Table S1). All these differentially expressed genes were defined as key genes related to the TLR pathway in CRC (DETLRgenes). Unsupervised hierarchical clustering analysis showed that the expression pattern of these DETLRgenes clustered all samples in the TCGA dataset into two groups with a significant association with sample disease status (*p* < 0.001, chi-squared test; Figure 1B).

### Construction and Characterization of lncRNA-Perturbed TLR-Signaling Network in CRC

**FIGURE 2:**
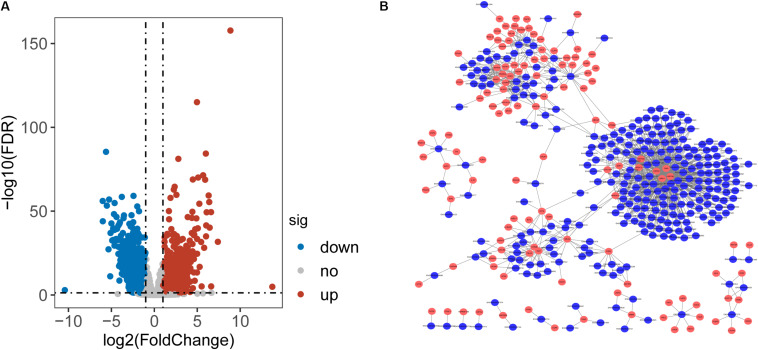
Construction and characterization of lncRNA-perturbed TLR-signaling network (LncTLRNet). **(A)** Volcano plot of the distribution of differentially expressed lncRNAs. **(B)** A global view of the LncTLRNet in CRC.

To identify lncRNAs associated with CRC, we performed differential expression analysis for 14799 lncRNAs between 41 paired CRC and control samples and identified 2154 differentially expressed lncRNAs (DElncRNAs) with [|log2(fold change]| > 1 and FDR-adjusted *p*-value < 0.05 (Supplementary Table S1). Among them, 933 lncRNAs were found to be up-regulated and 1221 down-regulated in CRC. Then we calculate the Pearson correlation coefficient between expression values of DETLRgenes and DElncRNAs in TCGA CRC patients. Those dysregulated lncRNA-mRNA pairs with PCC > 0.8 and *p* < 0.05 were considered as co-dysregulated lncRNA-mRNA crosstalks. Then all co-dysregulated lncRNA-mRNA crosstalks were integrated to form a global lncRNA-perturbed TLR-signaling network (LncTLRNet). As shown in Figure 2B, LncTLRNet included 402 nodes (280 lncRNAs and 122 mRNAs) and 982 edges.

### Identification of Prognostic TLR Pathway-Related lncRNAs Biomarkers in CRC

**FIGURE 3:**
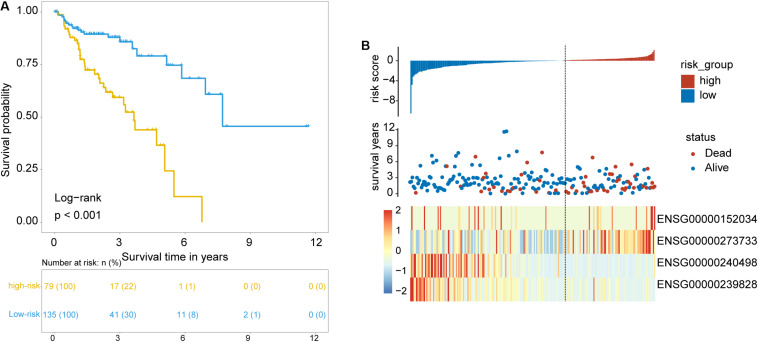
Identification of TLR pathway-related lncRNAs signature in the training set. **(A)** Kaplan-Meier survival curves of patients classified into high- and low-risk groups using the TLRLncSig. **(B)** The distribution of risk score, survival status, and lncRNA expression pattern.

To identify and validate the TLR pathway-related lncRNAs biomarkers in CRC, 427 CRC patients with survival information were divided equally into two groups: the training set (*n* = 214) and internal testing set (*n* = 213). Then we examined the association between 280 lncRNAs and 122 mRNAs in the LncTLRNet with overall survival (OS) using univariate and multivariate Cox regression analysis. A total of three TLR-related lncRNAs and one mRNA were significantly correlated with patients’ OS (Table 2). Finally, a TLR-related prognostic gene signature (TLRLncSig) was constructed based on the expression values of three TLR-related lncRNAs and one mRNA using the multivariate Cox regression coefficients as the weight, as follows: TLRLncSig = 17.134 × expression of *MCHR2* + 1.679 × expression of *AC011472.4* + −0.935) × expression of *CDKN2B* + (−36.272) × expression of *AC063944.1*. 214 patients of the training set were divided into the high-risk group and low-risk group according to the optimal cutoff value. As shown in Figure 3A, patients in the high-risk group had significantly shorter median survival time than those in the low-risk group (median OS 3.65 vs. 7.73 years, log-rank test *p* < 0.001). The five-year survival rate of patients in the high-risk group is 36.6%, whereas the corresponding rate is 79% in the low-risk group. Distribution of the lncRNA risk score, the survival status of the CRC patients, and the expression pattern of the TLRLncSig were shown in Figure 3B. Patients with high-risk scores tended to express high levels of *MCHR2* and *AC011472.4* in their tumors, whereas patients with low-risk scores tended to express high levels of *CDKN2B* and *AC063944.1* (Figure 3B).

**TABLE 2:** LncRNAs are significantly associated with overall survival in the signature.

Ensembl_id	Gene name	Genomic location	*P*-value^a^
ENSG00000152034	MCHR2	Chr 6: 99,918,519–99,994,247 (–)	0.030
ENSG00000273733	AC011472.4	Chr 19: 11,221,083–11,221,573 (+)	0.031
ENSG00000240498	CDKN2B	Chr 9: 21,994,139–22,128,103 (+)	0.023
ENSG00000239828	AC063944.1	Chr 3: 107,272,611–107,421,338 (–)	0.013

*^*a*^Derived form univariate analysis in the training set.*

### Validation of the TLRLncSig in the Testing Dataset

**FIGURE 4:**
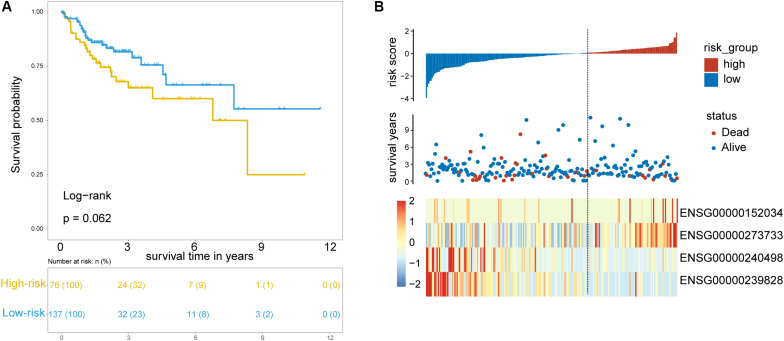
Validation of TLR pathway-related lncRNAs signature in the internal testing set. **(A)** Kaplan-Meier survival curves of patients classified into high- and low-risk groups using the TLRLncSig. **(B)** The distribution of risk scores, survival status, and lncRNA expression pattern.

To test the robustness of the TLRLncSig for prognosis prediction, the TLRLncSig was further validated in the testing dataset. By using the same risk score formula and cutoff value as for the training set, 213 patients of the testing set were classified into the high-risk group and low-risk group with significantly different OS (log-rank test *p* = 0.062; Figure 4A). As shown in Figure 4A, patients in the high-risk group had a significantly shorter median survival time than those in the low-risk group (median OS 8.33 vs. NA years). The five-year survival rate of patients in the high-risk group is 60.1%, whereas the corresponding rate is 66.4% in the low-risk group. Consistent with the findings in the training set, *MCHR2* and *AC011472.4* are risk factors which are overexpressed in high-risk patients, and *CDKN2B* and *AC063944.1* are protective factors which are overexpressed in low-risk patients (Figure 4B).

### Further Validation of the TLRLncSig in the Independent GSE39582 Set

**FIGURE 5:**
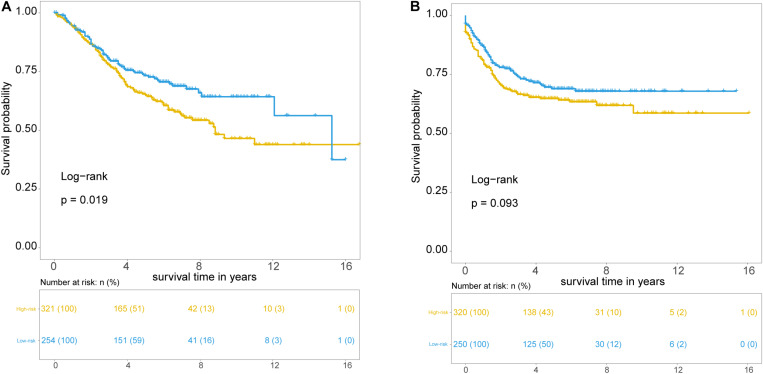
Independent validation of TLR pathway-related lncRNAs signature in the GSE39582 set. Kaplan-Meier survival curves of patients classified into high- and low-risk groups using the TLRLncSig for overall survival **(A)** and recurrence-free survival **(B)**.

Further validation of the TLRLncSig was conducted in the 585 patients of the GSE39582 set. However, gene expression data of the GSE39582 was profiled on Affymetrix HG-U133 Plus 2.0 platform, and only *MCHR2* and *CDKN2B* in the TLRLncSig were covered on the HG-U133 Plus 2.0 platform. Therefore, the TLRLncSig based only on these two TLR-related biomarkers without re-estimating parameters was applied to the independent GSE39582 set. Patients of the GSE39582 set were classified into the high-risk group (*n* = 254) and low-risk group (*n* = 321). As shown in Figure 5A, patients in the high-risk group had a significantly shorter median survival time than those in the low-risk group (median OS 8.83 vs. 15.25 years). The five-year survival rate of patients in the high-risk group is 64.9%, whereas the corresponding rate is 73.3% in the low-risk group. Furthermore, we also found that there are marginally significant differences in recurrence-free survival (RFS; log-rank test *p* = 0.093; Figure 5B).

### Independence of the TLRLncSig From Other Clinicopathological Factors

**FIGURE 6:**
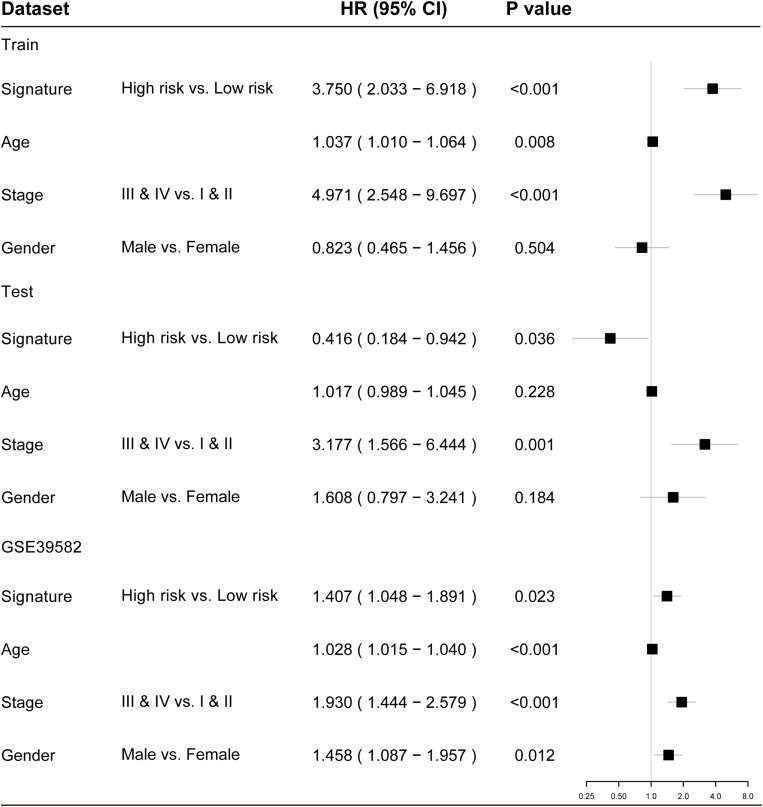
Forest plots showing multivariable Cox regression analyses in each data set.

**FIGURE 7:**
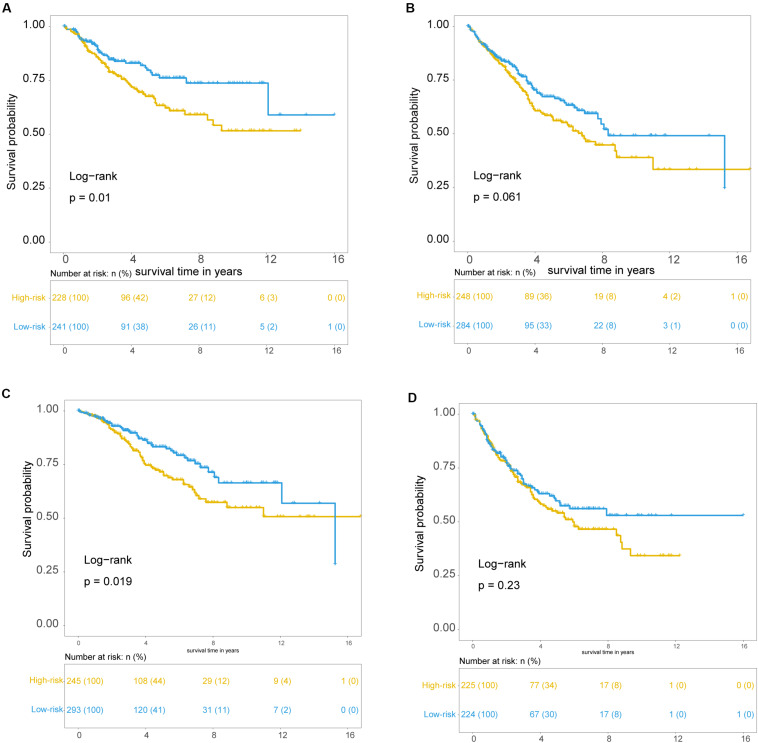
Independence of TLR pathway-related lncRNAs signature. Kaplan-Meier survival curves for patients within younger stratum **(A)** and an elder stratum **(B)**. Kaplan-Meier survival curves of patients in the early-stage **(C)** and late-stage **(D)**.

To examine whether the prognostic value of the TLRLncSig is independent of other clinicopathological factors of patients with CRC, we performed multivariate Cox regression analysis for clinicopathological factors (including age, stage, and gender), and the TLRLncSig. The results from the training set showed that the TLRLncSig (HR = 3.75; 95% CI 2.033-6.918, *p* < 0.001), age (HR = 1.037; 95% CI 1.010-1.064, *p* = 0.008), and stage (HR = 4.971; 95% CI 2.548-9.697, *p* < 0.001) were significantly correlated with OS of CRC patients (Figure 6). The TLRLncSig (HR = 0.416; 95% CI 0.184-0.942, *p* = 0.036) and stage (HR = 3.177; 95% CI 1.566-6.444, *p* = 0.001) were significant in the multivariate analysis (Figure 6). In the independent testing GSE39582 set, the TLRLncSig (HR = 1.407; 95% CI 1.048-1.891, *p* = 0.023), age (HR = 1.028; 95% CI 1.015–1.040, *p* < 0.001), stage (HR = 1.93; 95% CI 1.444–2.579, *p* < 0.001), and gender (HR = 1.458; 95% CI 1.087–1.957, *p* = 0.012) were significantly correlated with OS of CRC patients (Figure 6A). Therefore, we also performed a stratification analysis for age and stage. All patients were stratified into a younger stratum and an elder stratum. Within each age stratum, the TLRLncSig could further subdivide CRC patients into the high-risk group and low-risk group with significantly different OS (Figures 7A,B). All patients were further stratified into an early-stage stratum and a late-stage stratum. When the TLRLncSig was applied to the early -stage stratum, the TLRLncSig could classify stage early-stage patients with OSCC into high- and low-risk groups with significantly different OS (log-rank *p* = 0.019; Figure 7C). For late-stage patients, although the *p*-value was 0.23, which above the 0.05 level, the high-risk patients were observed to have shorter survival than those in the low-risk group (Figure 7D). The results of the multivariable analysis thus indicated that the TLRLncSig is independent of other clinicopathological factors for survival prediction of CRC patients.

## Discussion

It has been reported that CRC, like numerous other solid tumors, is a heterogeneous and molecularly complex disease characterized by different molecular and phenotypic characteristics (Fanelli et al., 2020). Although the classic staging system could classify CRC patients into different prognostic groups based on the extent of the primary tumor, regional lymph nodes, and the presence/absence of distant metastases (Punt et al., 2017), it is insufficient for patients with the same clinical features. Advances in molecular omics studies have demonstrated that molecular expression alteration can perturb the key signaling network contributing to tumorigenesis and cancer therapeutics. TLR-signaling pathway is a well-known player in inflammation, immune cell regulation, survival, and proliferation (Kawai and Akira, 2010; Li et al., 2010). Many studies have shown that lncRNAs play critical roles in various biological processes by regulating protein-coding genes at the transcriptional level, post-transcriptional, and epigenetic levels (Kornienko et al., 2013). Several lncRNAs (such as *lincRNA-Cox2,CRNDE, and* HIX003209) have recently been identified to be involved in regulating TLR signaling and innate immune (Murphy and Medvedev, 2016), which highlighted the roles of lncRNAs in TLR signaling network. To explore the functional roles of the lncRNA-mediated TLR-signaling network, we first performed compared expression pattern of lncRNAs and TLR-related genes between CRC and normal samples and identified aberrant lncRNAs and TLR genes involved in CRC development. By using a multi-step computational approach, we constructed a global lncRNA-perturbed TLR-signaling network by focusing on these deregulated lncRNAs and TLR genes and their co-expression relationship. The resulting lncRNA-perturbed TLR-signaling network contained 280 lncRNAs and 122 TLR genes, implying that abnormal expression of these 280 lncRNAs might perturb the TLR-signaling network which contributed CRC development and progression.

A large number of studies have indicated that lncRNAs are an essential component in cancer biology by acting as oncogenic and tumor-suppressive factors, and have become emerging and suitable diagnostic and prognostic biomarkers or therapeutic targets in various cancers including CRC (Gibb et al., 2011; Cheetham et al., 2013; Kornienko et al., 2013; Zhu et al., 2015; Zhou et al., 2015b, 2017, 2018b, 2020; Zhong et al., 2018; Wang et al., 2019; Zhang et al., 2019; Sun et al., 2020). Therefore, we further explored the prognostic value of these 280 TLR-related lncRNAs in CRC patients. By performing univariate and multivariate Cox regression analysis, we identified three lncRNAs (*MCHR2, AC011472.4*, and *AC063944.1*) from the list of 280 TLR-related lncRNAs and one mRNA (*CDKN2B*) that are significantly and independently associated with OS of CRC patients in the training set. To facilitate the clinical application, there four lncRNAs biomarkers were integrated into a lncRNAs signature, which classified the CRC patients of training set into two groups with significantly different OS. Furthermore, the prognostic value of the TLRLncSig was further validated in the TCGA internal testing set profiled by the RNA-seq platform and another GEO patient set profiled by microarray platform, demonstrating the robustness and cross-platform performance of the TLRLncSig in predicting OS for CRC patients. Although some limitations exist, the TNM staging system still is most commonly used nowadays for risk stratification assessment in the clinical application. Therefore, we further examine whether the prognostic value of the TLRLncSig is independent of known clinicopathological factors in each dataset. By performing multivariable Cox regression analysis and stratification analysis, the TLRLncSig also revealed significant prognostic performance after adjusted by known clinicopathological factors. These results suggested that the predictive value of the TLRLncSig is independent of known clinicopathological factors.

Although thousands of lncRNAs have been discovered and recorded in public databases, most of them are not well functionally characterized. After literature mining, three identified novel TLR-related lncRNAs were not reported and studied. Therefore, further experimental studies on these three lncRNAs should be conducted to gain functional insights into the TLR signaling network. Another limitation of this study is that the prognostic value of the TLRLncSig was validated in two CRC patient sets. Therefore, the generalizability of the TLRLncSig should be further verified in prospective CRC datasets.

In conclusion, the present study, for the first time, investigated the potential involvement of lncRNAs in the TLR signaling network by constructing and characterizing lncRNA-perturbed TLR-signaling network and identified a novel TLR-related signature as a potential genomic tool for prognosis risk assessment. Our study provides novel insights into the lncRNAs-mediated TLR-signaling network and reveals the potential roles of lncRNAs in CRC development and progression. Investigations into the molecular mechanisms of lncRNAs in TLR-signaling network and their clinical significance warrant future work TLR-signaling network.

## Data Availability Statement

Publicly available datasets were analyzed in this study. This data can be found here: CRC datasets used in this study were obtained from the UCSC Xena platform (https://xena.ucsc.edu/) and GSE39582 (https://www.ncbi.nlm.nih.gov/geo/query/acc.cgi?acc~=~GSE39582).

## Author Contributions

DZ and FP conceived and designed the experiments. YC, ZL, JL, and LY analyzed the data. YC wrote the manuscript. All authors read and approved the final manuscript.

## Conflict of Interest

The authors declare that the research was conducted in the absence of any commercial or financial relationships that could be construed as a potential conflict of interest.

## Footnotes

1 https://xena.ucsc.edu/

2 https://www.ncbi.nlm.nih.gov/geo/query/acc.cgi?acc=GSE39582

3 https://www.gencodegenes.org/

4 https://string-db.org/

## Supplementary Material

TABLE S1 Lists of differentially expressed lncRNAs and TLR-related genes between 41 paired CRC and control samples.
